# High-efficiency second-order nonlinear processes in an optical microfibre assisted by few-layer GaSe

**DOI:** 10.1038/s41377-020-0304-1

**Published:** 2020-04-17

**Authors:** Biqiang Jiang, Zhen Hao, Yafei Ji, Yueguo Hou, Ruixuan Yi, Dong Mao, Xuetao Gan, Jianlin Zhao

**Affiliations:** MOE Key Laboratory of Material Physics and Chemistry Under Extraordinary Conditions and Shaanxi Key Laboratory of Optical Information Technology, School of Physical Science and Technology, Northwestern Polytechnical University, Xi’an, 710129 China

**Keywords:** Nonlinear optics, Fibre optics and optical communications

## Abstract

The centrosymmetric nature of silica fibre precludes the realisation of second-order nonlinear processes in optical fibre systems. Recently, the integration of 2D materials with optical fibres has opened up a great opportunity to develop all-fibre active devices. Here, we demonstrate high-efficiency second-order nonlinear frequency conversions in an optical microfibre assisted with few-layer gallium selenide (GaSe) nanoflakes. Attributed to the strong evanescent field of the microfibre and ultrahigh second-order nonlinearity of the GaSe nanoflakes, second harmonic generation (SHG) and sum-frequency generation (SFG) are effectively achieved with only sub-milliwatt continuous-wave (CW) lasers in the wavelength range of 1500–1620 nm, covering the C and L telecom bands. The SHG intensity from the microfibre is enhanced by more than four orders of magnitude with the assistance of the GaSe nanoflakes on fibre nonlinear processes. Moreover, in the SFG process, the intensity transfer between different frequencies can be effectively manipulated by changing the wavelengths and powers of two pump lasers. The realised strong second-order nonlinearity in the GaSe-integrated microfibre might expand the applications of all-fibre devices in all-optical signal processing and new light source generation at awkward wavelengths.

## Non-linear optics: All-fibre optical wavelength converter

An optical fibre coated with gallium selenide nanoflakes could convert the wavelength of an incident light efficiently via the second-order nonlinear optical effect. This wavelength-converting optical fibre, developed by Xuetao Gan and colleagues at China’s Northwestern Polytechnical University, could be incorporated into existing optical fibre-based infrastructure to provide a wide range of applications, including in telecommunications, signal processing and multi-parameter sensing. The researchers found that, with the assistance of gallium selenide nanoflakes, the efficiency of wavelength conversion in the coated microfibre was more than four orders of magnitude higher than that obtained in a bare microfibre. The all-fibre wavelength converter allows the easy operation with a sub-milliwatt continuous-wave laser over a broad wavelength range, and has the considerable stability and repeatability.

## Introduction

Silica optical fibres exhibit intrinsic features such as ultralow loss, a high damage threshold, and a small mode field, enabling the possibility of long-haul communications and sensing. The advance of optical fibres also gives birth to nonlinear fibre optics due to the long interaction length and high power density in the fibre core. However, the centrosymmetric and amorphous properties of silica fibres preclude the possibility of second-order nonlinear processes, and the majority of studies and applications are based on its third-order nonlinearity^1^, such as modulation instability^2^, temporal solitons^3,4^, ultrashort pulse mode locking^5^, supercontinuum generation^6,7^, third-harmonic generations (THGs)^8,9^, frequency combs^10^, etc. Compared with common nonlinear crystals, optical fibres have small nonlinear susceptibility *χ*^*(*3)^, making a high pump power essential in third-order nonlinear processes. This limits the applications of fibre technology and presents challenges to power consumption in the whole fibre system. In nonlinear optics, second-order nonlinear responses are the primary alternative, relying on much higher second-order nonlinear susceptibility *χ*^*(*2)^ (i.e., higher than *χ*^*(*3)^) for optical frequency conversions, electro-optical effects, etc. Unfortunately, limited by the centrosymmetry of silica, there is no second-order nonlinear response in optical fibres. Although the material defects and fibre surface may induce weak second-order nonlinearity, the typical second-order nonlinear processes require enormously high peak power, on the order of 10^4^ watts^11–18^.

To facilitate the second-order nonlinearity of a fibre, a variety of techniques, such as thermal poling^19–23^, electric field poling^24,25^, and optical poling^18^ of these special fibres, have been employed to artificially break the centrosymmetry. These special fibres require relatively complex processes and harsh fabrication conditions, including a high poling voltage above the kilo-volt level, high temperatures over 200 °C, or high-intensity femtosecond laser pulses, for effective poling. Nevertheless, pulsed pump lasers with high peak powers are still indispensable, as their second-order nonlinearity can be exploited. Alternative solutions would hence be welcome to avoid delicate fibre post-processing while still allowing operations over a wide pump wavelength range.

In this work, we demonstrate the realisation of high-efficiency second-order nonlinear processes in a microfibre by integrating few-layer gallium selenide (GaSe) nanoflakes. Few-layer GaSe is chosen because it has strong second-order nonlinearity^26–29^ and promises efficient optical frequency conversion ranging from the visible to the terahertz wavelength range^30^. Second-order nonlinear processes, including second harmonic generation (SHG) and sum-frequency generation (SFG), are achieved in the all-fibre structure with the pumps of different continuous-wave (CW) lasers, arising from the strong interaction between few-layer GaSe flakes and the microfibre evanescent field. The use of microfibres can offer long and controllable interaction lengths for comparable acceptance bandwidths. By examining the relaxed phase-matching condition with the pump wavelengths and microfibre parameters, strong SHG signals can be obtained in a wide wavelength range (1500–1620 nm) covering the whole C and L telecom bands as well as the O band (1310 nm). The widely tuneable SHG enables us to excite the SFG signal as well. The proposed all-fibre frequency converter, with the assistance of atomic layered GaSe with high nonlinearity, may open up a novel way to achieve high-performance all-fibre nonlinear devices.

## Results

**Fig. 1:**
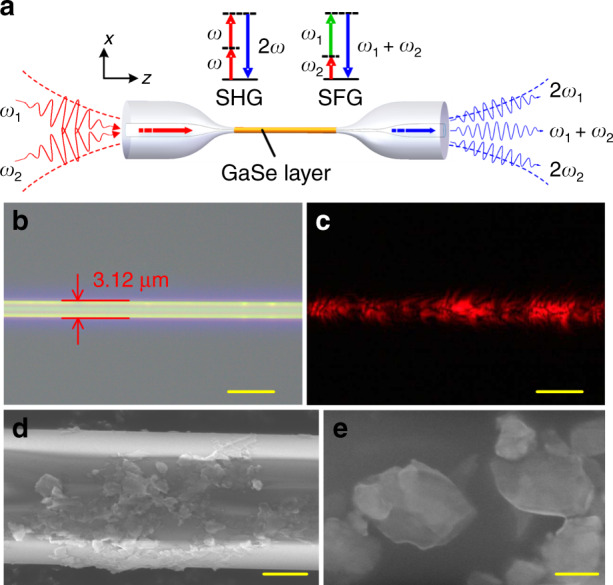
Structure and characterizations of the microfibre integrated with few-layer GaSe. **a** Schematic of the operations of SHG and SFG from the GaSe-integrated microfibre. Top inset shows the energy diagrams of the SHG and SFG in the fibre device. **b**, **c** Optical microscopic images of the GaSe-integrated microfibre under bright and dark fields when launched by a 635 nm laser. **d**, **e** Scanning electron microscope images of the GaSe-integrated microfibre at different magnifications. Scale bars: **b**, **c**: 5 μm; **d**: 1 μm; **e**: 100 nm

The operations of second-order nonlinear processes of the GaSe-integrated microfibre are schematically illustrated in Fig. 1a. A microfibre pulled from the standard single mode fibre is employed to provide a strong evanescent field (Supplementary Information), which can interact with the integrated 2D GaSe nanoflakes effectively. The effective interaction length can be adjusted by controlling the region of the GaSe deposition. Pumped by two CW lasers, the SHG and SFG can be excited from the GaSe nanoflakes. Part of the frequency up-conversion signal couples to the microfibre and forms a guiding mode to propagate in the microfibre.

Figure 1b displays the optical microscopic image of a fabricated device, which shows a microfibre diameter of ~3.1 μm. To feature the integrated GaSe nanoflakes, a red laser is launched into the fibre device, and the light scattered from the GaSe-integrated microfibre is collected from the top using an optical microscope, as shown in Fig. 1c. To evaluate the insertion loss caused by the scattering and absorption^29,30^, we measure the transmission spectra of the microfibre before and after GaSe integration, showing an average loss of less than 1 dB (Supplementary Fig. S1). The oscillations of the spectra, which originate from the multimode propagation and their modal interference in the microfibre, are observed in the shorter wavelength region^31–33^. In the device fabrication, to reduce the optical loss induced by the GaSe deposition, a dilute dispersion with GaSe nanosheets, a pipette with a micro-volume, and multiple depositions are adopted to avoid excessive overlapping and agglomeration of the nanoflakes (for details, see the Supplementary Information). Fig. 1d, e display the morphology of the GaSe-integrated microfibre characterised by using a scanning electron microscope (SEM). As shown in the SEM images at different magnifications, the few-layer GaSe flakes are tightly wrapped around the microfibre surface. The GaSe nanoflakes are also characterised by an atomic force microscope and a transmission electron microscope to examine their uniformity and thickness (see Supplementary Fig. S3). The average thickness of the GaSe nanoflakes is ~4.1 nm, and the size of the nanoflakes is in the range of 0.05–0.5 μm. The employed ε-GaSe with the noncentrosymmetric point group *D*_*3h*_ has second-order nonlinearity with any arbitrary layer. Thicker GaSe nanoflakes can assist in improving the SHG efficiency, though at the expense of scattering loss of the microfibre guiding modes. Taking into account the loss and SHG efficiency, the ease of operation at the average thickness of a few nanometres makes it possible to effectively excite the SHG signals in the experiment.

**Fig. 2:**
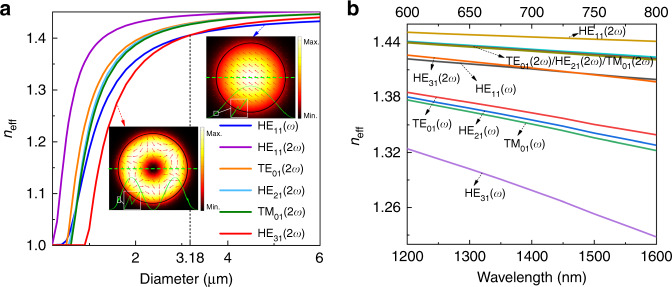
Numerical calculation of effective refractive indices of modes in the GaSe-integrated microfibre hybrid waveguide. **a** Effective refractive index as a function of the microfibre diameter for the fundamental wave (*ω*) at 1550 nm and possible SH waves (2*ω*) at 775 nm. The phase-matching condition is satisfied with a microfibre diameter of approximately 3.18 μm. Insets are the modal distributions of two phase-matched modes HE_11_(*ω*) and HE_31_(2*ω*) in the microfibre (the GaSe layer is indicated by the black lines). The amplitudes of the mode fields along the radial direction are indicated by the green lines. **b** Dispersions of possible modes in the hybrid waveguide. Top and bottom coordinates represent the wavelengths of the fundamental wave (*ω*) and SH wave (2*ω*), respectively

To explain the designed diameter of the microfibre and possible mode matching, we built a theoretical model of the GaSe-integrated microfibre hybrid waveguide to study the optimal phase-matching condition. The thickness of the GaSe layer is set at the measured value of 4.1 nm. Multimode propagation is possible in the microfibre, especially below the cutoff wavelength. To realize efficient frequency up-conversion, it is necessary to match the effective indices of the fundamental wave (*ω*) and SH wave (2*ω*)^34–36^. Taking the pump wavelength of 1550 nm as an example, Fig. 2a depicts the effective refractive index (*n*_eff_) of possible modes in the hybrid waveguide. Clearly, we demonstrate the achievement of effective refractive index matching by engineering the microfibre diameter. The phase matching condition can be satisfied for a higher-order mode HE_31_(2*ω*) at 775 nm and the fundamental mode HE_11_(*ω*) at 1550 nm when the microfibre diameter is 3.18 μm. The two insets of Fig. 2a show the corresponding mode distributions in the hybrid waveguide with the matched diameter. From the radial distribution, the mode field is extended to the microfibre-GaSe boundary, and there is an abrupt enhancement in the GaSe layer, as shown in the enlarged region of the inset. The amplitudes of the evanescent fields of HE_11_(*ω*) and HE_31_(2*ω*) distributed in the 4.1 nm thick GaSe flake are 0.137% and 0.17% of the total mode field, respectively. It is noted that in the aforementioned experiment, the diameter of the fabricated microfibre is ~3.1 μm, which is very close to the optimal value for perfect phase matching. Additionally, as shown in Fig. 2a, there are other possible matching points between the fundamental wave and SH waves in the smaller diameters below 1 μm. In the experiment, only the diameter of ~3.18 μm for the phase-matching condition was taken into account for ease of fabrication and to achieve a relatively robust structure. Moreover, we set the diameter of the microfibre to the actual size and calculated the dispersion relationship of possible modes in the wavelength range of 1200–1600 nm. The results are shown in Fig. 2b, and the top and bottom coordinates correspond to the wavelengths of the fundamental waves and SH waves, respectively. In this hybrid waveguide, the effective refractive indices of the fundamental wave HE_11_(*ω*) and SH wave HE_31_(2*ω*) are very close in a wide range, which relaxes the phase-matching requirement and provides the possibility for SHG in a wide operation range.

**Fig. 3:**
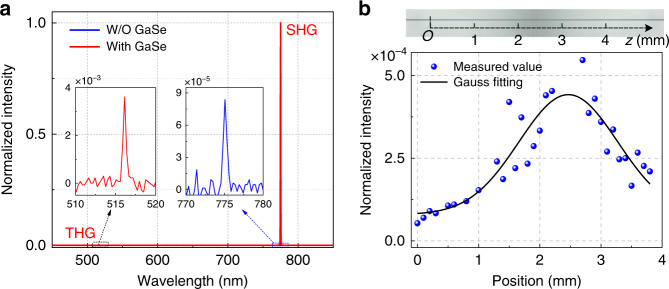
Frequency up-conversion signals collected from the GaSe-integrated microfibre. **a** Frequency up-conversion spectra from the microfibre with and without GaSe integration when pumped by a pulsed laser at 1550nm, showing SHG and THG peaks at 775 nm and 516.7 nm, respectively. The left inset is the enlarged THG spectrum from the GaSe-integrated microfibre, and the right inset is the enlarged SHG spectrum from the bare microfibre. Normalised intensities of the SHG peaks are 1 and 8.4 × 10^−5^, respectively; in comparison, the SHG intensity is enhanced by more than four orders of magnitude after GaSe integration. **b** Spatial distribution of the SHG signal side-collected from the surface along the microfibre, indicating an effective interaction length of more than 4 mm. Top inset shows an optical microscopic image of the microfibre

A pulsed laser with a high peak power is first employed to tentatively examine the frequency up-conversion signals from the GaSe-integrated microfibre (the detailed experimental arrangement is shown in Supplementary Fig. S3). Fig. 3a shows a typical frequency up-conversion spectrum of the device when pumped by a 1550 nm pulsed laser. There are two peaks, observed at 775 nm and 516.7 nm, corresponding to the converted wavelengths of the SHG and THG signals of the pumped laser. As a comparison, the frequency up-conversion signal from the microfibre before GaSe integration is also examined under the same experimental conditions. Arising from the highly confined modes of the microfibre and the symmetry breaking at the microfibre-air interface^14^, a weak SHG peak at 775 nm is observed from the bare microfibre, as shown in the right inset of Fig. 3a. The SHG from the GaSe-integrated microfibre is more than four orders of magnitude stronger than that from the bare microfibre. This could be attributed to the ultrahigh second-order nonlinearity of the GaSe nanoflakes and effective light-matter interactions via the microfibre evanescent field^26,28,37^, which also implies the assistance of the GaSe nanoflakes in the second-order nonlinear responses of the fibre devices. When the diameter of the microfibre deviates from the designed optimal value, the SHG signal will be much weaker (shown in Supplementary Fig. S4) due to the increase in phase mismatch.

To verify the origin of the strongly enhanced SHG, in addition to measuring the SHG signals transmitting through the microfibre, we also implement measurements that involve collecting the signal scattered from the side of the GaSe-integrated microfibre using a vertical microscope setup. A detailed sketch of the measurement setup is illustrated in Supplementary Fig. S5. When a pulsed laser propagates in the microfibre, SHGs of the GaSe nanoflakes excited by the microfibre evanescent field will partly scatter outwards perpendicular to the microfibre surface, which can be collected by the objective lens of the microscope. The GaSe-integrated microfibre is mounted on a piezo-driven micro-displacement platform to monitor the spatial position dependence of the vertically scattered SHG signals. The top image of Fig. 3b shows the optical microscopic image of the device. The SHG signals are collected from different locations along the microfibre (*z*-axis), and the results are shown in the bottom image of Fig. 3b. The side-collected SHG signal depends on the scattering at the local position, which is determined by the interaction between the evanescent field and the local distribution of the GaSe nanoflakes. Consequently, the maximum intensity of the SHG signal appears in the vicinity of the microfibre waist due to a strong evanescent field and densely distributed GaSe nanoflakes. An effective interaction length of more than 4 mm is indicated. Comparing the SHG signals in Fig. 3a, b, the intensity from the microfibre is more than 3 orders of magnitude greater than that collected only from a local position on the surface, owing to the cumulative results of the SHG in the microfibre. It therefore indicates a higher collection efficiency in this all-fibre structure, which could be further enhanced by choosing the optimal diameter for effective mode coupling.

**Fig. 4:**
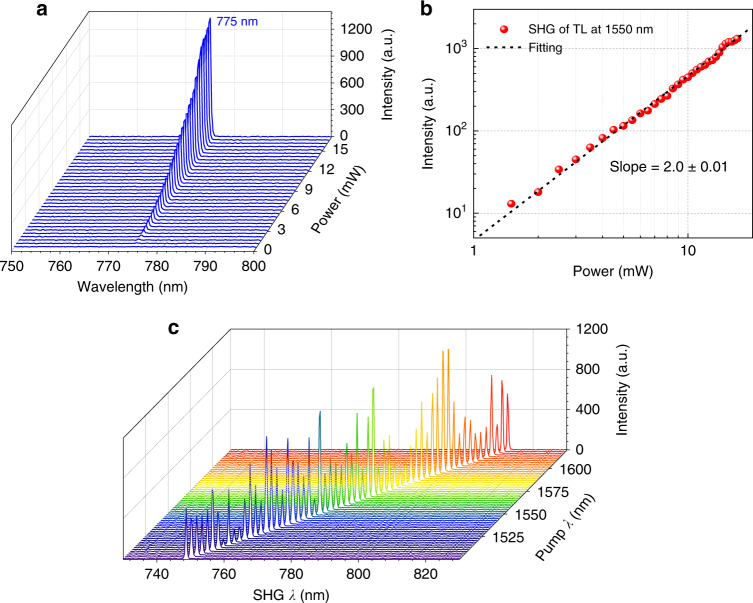
Pump-power- and wavelength-dependence of SHG pumped by a tuneable CW laser. **a** Measured spectral evolution of SHG when the wavelength of the tuneable laser is fixed at 1550 nm and the power is varied from 0 to 17 mW. **b** Corresponding log-log plot of the SHG intensity versus the incident power, with a fitting slope of 2.0 ± 0.01. **c** Spectral evolution of SHG as the pump wavelength changes from 1500 nm to 1620 nm

In practical applications, CW-pumped nonlinear fibre optics with simple, low-power and low-cost light sources, such as semiconductor laser diodes, would be highly desirable. The high-efficiency SHG from the GaSe-integrated microfibre offers the possibility of CW operation. To verify this, CW lasers at two important telecom wavelengths, i.e., 1550 nm and 1310 nm, are employed to independently pump the SHG, and the results are shown in Fig. 4. Strong SHG signals at 775 nm are observed. To further evaluate the dependence of the SHG intensity on the pump power, we adjusted the incident power from 0 to 17 mW at a fixed wavelength of 1550 nm. From the spectral evolution of the SHG signal shown in Fig. 4a, the SHG intensity clearly increases as the pump power increases. Here, the pump power is the optical power incident into the GaSe-integrated microfibre. The detailed dependence is log-log plotted in Fig. 4b. The fitting slope of 2.0 ± 0.01 reveals a quadratic dependence on the pump power, followed by electric dipole theory^26^. At another pump wavelength of 1310 nm, we obtain a similar spectrum evolution (see Supplementary Fig. S6). With a pump power of 8 mW launched into the GaSe-microfibre, the collected SHG power from the output side of the GaSe-microfibre is approximately 185 pW, measured with a photomultiplier tube. The SHG conversion efficiency is estimated as 185 pW/(8 mW)^2^ = 2.9 × 10^−4^ %/W. This efficiency is more than four orders of magnitude higher than those obtained in the previously reported works on SHG in fibres (see the Supplementary Information), benefiting from the assistance of the strong second-order nonlinearity of GaSe.

We also evaluate the pump wavelength dependence of the SHG by changing the tuneable laser wavelength, as shown in Fig. 4c. The SHG can be clearly observed in the wavelength range of 1500–1620 nm, covering the important C + L telecom band (1525–1620 nm). Meanwhile, the SHG intensity from the GaSe-integrated microfibre fluctuates with the pump wavelength due to the multimode propagation and interference in the short wavelength range (see Supplementary Fig. S1) and the global modulation by the phase-matching condition. In ordinary single-mode fibres (SMFs) for the telecom band, only the fundamental mode HE_11_ can propagate in the fibre core with low loss, since the light is confined by the relatively thick cladding. However, for the microfibre in air, the mode field is extended to the microfibre-external medium interface, even to the air (see the inset of Fig. 2a), and the large refractive index difference in the microfibre/air waveguide enables multimode propagation in the microfibre region, leading to intermodal interference as well as wavelength-dependent loss (shown in Supplementary Fig. S1). Therefore, this widely tuneable SHG in the GaSe-integrated microfibre can also be modulated by the microfibre diameter and length, which affect the propagation of the intermodal interference.

**Fig. 5:**
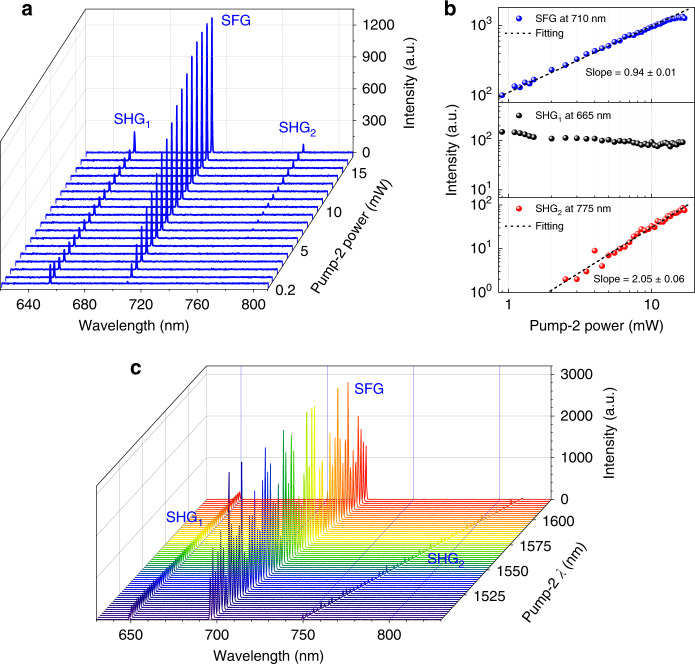
Multiple frequency conversions from the GaSe-integrated microfibre. **a** Measured spectral evolution of SHG_1_, SHG_2_ and SFG when pumped by two CW lasers at 1310 nm (Pump-1) and 1550 nm (Pump-2). **b** Log-log plots of the power dependences of SFG (top), SHG_1_ (middle) and SHG_2_ (bottom) obtained by varying the incident power of Pump-2. **c** Spectral evolution of SHG_1_, SHG_2_ and SFG with variation of the Pump-2 wavelength from 1500 nm to 1620 nm

The achieved widely tuneable CW-pumped SHG promises the implementation of other second-order nonlinear processes as a consequence of the exemption of the accurate synchronisation of multiple pulsed lasers. To verify this possibility, we use two CW lasers to simultaneously pump the GaSe-integrated microfibre. A distributed feedback laser, i.e., Pump-1, has a fixed wavelength of 1310 nm and a stable output power of 20 mW, and a tuneable narrowband laser, i.e., Pump-2, has various wavelengths and powers to investigate the wavelength- and power dependence of the frequency conversions. Fig. 5 displays the measured spectra of multiple frequency conversions from the GaSe-integrated microfibre. SHG peaks pumped by the two CW lasers appear at *λ*_SHG1_ ~ 655 nm and *λ*_SHG2_~ 775 nm. Between them, there is another peak at ~710 nm, corresponding to the SFG peak of the two pump lasers according to the frequency (or wavelength) calculations. Meanwhile, by increasing the output power of Pump-2 at 1550 nm, the SFG (*ω*_1_ + *ω*_2_) and SHG_2_ (2*ω*_2_) gradually become stronger, while the SHG_1_ (2*ω*_1_) signal experiences a slight decrease. In the process of SHG_1_ (or SHG_2_), only Pump-1 at 1310 nm (or Pump-2 at 1550 nm) participates, and two photons of this fundamental wave are converted into one frequency-doubled photon. Hence, the SHG_2_ signal intensity is a quadratic function of the pump power, with a fitting slope of 2.05 ± 0.06 from the experimental results shown in the bottom curve of Fig. 5b. In the SFG process, both pump lasers are employed to generate the frequency-summed photons. Pump-2 contributes only one photon to the SFG process, and then the log-log plot of the power dependence has a slope of 0.94 ± 0.01 (close to 1), as indicated in the top curve of Fig. 5b. However, since the SFG converts most of the photon energy of the fundamental waves, the intensities of the SHG_1_ and SHG_2_ signals are much weaker than those pumped independently by the individual CW laser. This also explains the gradually decreased intensity of SHG_1_.

Similarly, we evaluate the wavelength dependence of the SFG by fixing the Pump-1 wavelength at 1310 nm and scanning the Pump-2 wavelength from 1500 nm to 1620 nm, as shown in Fig. 5c. There is a stable SHG_1_ signal at 655 nm and gradually red-shifted SHG_2_ and SFG signals as the Pump-2 wavelength increases. Additionally, the SHG and SFG intensities fluctuate due to the intermodal interference in their multimode propagations. Clearly, the intensity of SFG involving both pump lasers is above one order of magnitude greater than the two SHG signals arising from two-photon conversions with only one pump laser. Therefore, more photon energy of the fundamental waves will transfer to the sum-frequency photon in a wide wavelength range.

In addition, the output SHG signals of the GaSe-integrated microfibre at different times are detected to determine the stability and pump power threshold of the frequency conversion (for details, see Supplementary Fig. S7). The multiple measurement results show a stable SHG signal within an ~9% fluctuation, which could be caused by a disturbance such as air flow, vibration and dust absorption. The stability can be further improved by effective packaging for engineering applications. Meanwhile, by reducing the power of the pump lasers to observe the detectable SHG and SFG signals, the thresholds can be as low as l mW and 0.2 mW for SHG and SFG, respectively. Moreover, the microfibre, space-to-fibre coupling and other insertion losses are taken into account, totally tested by ~4 dB (Supplementary Fig. S1); hence, much lower power, even tens of microwatts, can generate the above detectable frequency conversion signals.

## Discussion

In summary, we report the first achievement of greatly enhanced second-order nonlinear processes (SHG and SFG) under nonresonant excitation conditions in a GaSe-integrated microfibre hybrid waveguide. Benefitting from the ultrahigh second-order nonlinear susceptibility *χ*^(2)^ of few-layer GaSe, the measured SHG from the GaSe-integrated microfibre is enhanced by more than four orders of magnitude in comparison with the SHG from a bare microfibre. Further power- and wavelength-dependences reveal that the efficient SHG can be excited with only a sub-milliwatt CW laser over a large range of wavelengths (hundreds of nanometres) attributed to relaxed phase-matching conditions and nonresonant excitation. This widely tuneable CW-pumped SHG allows us to implement another second-order nonlinearity (SFG) in a wide operation range owing to the exemption of synchronisation of the two pulsed lasers. The SHG and SFG from the device also have relatively high stability and repeatability. The efficiency of the frequency conversion can be further enhanced by using a direct chemical vapour deposition (CVD) growth technique for the perfect coating of 2D materials, which could facilitate a strong and tuneable light-matter interaction^15,38^. The proposed CW pumped all-fibre frequency converter is easy to integrate with the current telecom infrastructure and thus has extensive applications in frequency-converted fibre laser sources, all-optical signal processing, fibre sensors, and so on. Additionally, we believe that this hybrid fibre waveguide device, by integrating other atomic layered materials, can pave the way for achieving high-performance frequency modulation and manipulation in an all-fibre structure.

## Materials and methods

### Device fabrication and characterisation

The employed microfibre is pulled by the flame brushing technique^39^. The flame size and temperature and the pulling speed are controlled to obtain a uniform microfibre with a diameter of ~3.1 μm over a length of several millimetres. To facilitate the integration of the GaSe material with the microfibre, the few-layer GaSe nanosheets produced by the massive liquid exfoliation method are dispersed in a water-alcohol mixture^40^. A photothermal deposition technique is utilised to deposit few-layer GaSe nanosheets onto the microfibre region. We use an atomic force microscope and a transmission electron microscope to examine the uniformity, thickness, microstructure and chemical composition of the employed few-layer GaSe nanosheets.

### Modelling and simulations

We use the COMSOL Multiphysics software to build a theoretical model of the GaSe-integrated microfibre hybrid waveguide and simulate the possible modes and their effective indices in the hybrid waveguide. Then, we obtain the phase-matching condition at the optimal microfibre diameter and selected operation wavelength range in the numerical simulation.

## Supplementary information

Supplementary Information
