# Supplementary material for: High-efficiency second-order nonlinear processes in an optical microfibre assisted by few-layer GaSe
Source: Light Sci Appl. 2020 Apr 17;9:63. doi: 10.1038/s41377-020-0304-1 (PMC7165163; doi:10.1038/s41377-020-0304-1)
Supplement: Supplementary file 1 — Supplementary Information [file 41377_2020_304_MOESM1_ESM.pdf]

Supplementary Information for

High-efficiency second-order nonlinear processes in an optical microfibre assisted by few-layer GaSe

Biqiang Jiang, Zhen Hao, Yafei Ji, Yueguo Hou, Ruixuan Yi, Dong Mao, Xuetao Gan*, and Jianlin Zhao*

MOE Key Laboratory of Material Physics and Chemistry Under Extraordinary Conditions and Shaanxi Key Laboratory of Optical Information Technology, School of Physical Science and Technology, Northwestern Polytechnical University, Xi'an 710072, China

E-mail: xuetaogan@nwpu.edu.cn; jlzhao@nwpu.edu.cn

Fabrication and characterization of GaSe-integrated microfibre

The employed microfibre was pulled by flame brushing technique [1], and a uniform diameter of ~3.1 μm over a length of several millimetres was obtained by carefully controlling the flame size and temperature as well as the pulling speed. The ε-polytype of GaSe is chosen because it promises efficient optical frequency conversion ranging from visible to terahertz wavelength [2]. Among the large family of 2D materials, the stacking sequence of ε-GaSe induces the absence of inversion center for arbitrary layer thickness, and then the observations of SHG in the layered GaSe itself indicates stronger signal that is 1~2 orders of magnitude larger than from monolayer MoS_2 under the same excitation power [3-5]. Also, the employed few-layer GaSe nanosheets were fabricated by a simple and effective liquid exfoliation method [6].

The few-layer GaSe nanosheets dispersed in water-alcohol mixture with the concentration of ~1.0 $mg \cdot ml^{-1}$ were deposited onto the waist region of microfibre by using a dip-coating technique assisted with a red light source. During the deposition process, to reduce the optical loss, it is essential to relieve the isolated agglomerations and make the GaSe nanosheets form relatively uniform films wrapped around the microfibre. We used a pipette with micro-volume to drop the dilute dispersion of GaSe nanosheets onto the microfibre by multiple drop-coatings. After each deposition, the microfibre with GaSe nanoflakes was dried in the air for 30 minutes before the next drop-coating of the GaSe nanoflakes. This procedure could avoid the excessive overlapping and agglomeration of the nanoflakes and effectively control the uniformity and thickness of the deposited GaSe film. In the meantime, the transmission of the microfibre was monitored to evaluate the induced loss and recorded by an optical spectrum analyzer. Since the GaSe layer has a lower absorption coefficient in visible (especially longer than 620 nm) and near-infrared regions [2,7], the measured transmission spectra before and after GaSe integration show the average loss of less than 1 dB, as shown in Fig. S1a. Also from Fig. S1b, the fluctuation of the spectra were observed in the shorter wavelength region, which originates from the multimode propagation and their modal interference in the microfibre [8-10].

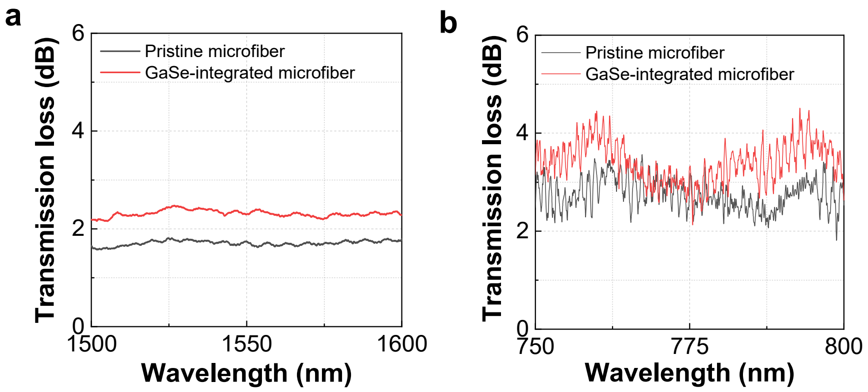

Fig. S1 Transmission loss of the GaSe-integrated microfibre in comparison with that of a pure microfibre in (**a**) fundamental wave (1550~1600 nm) and (**b**) SHG (750~800 nm) wavelength regions.

The uniformity and thickness of the employed few-layer GaSe nanosheets for the microfibre integration were examined by using an atomic force microscope (AFM), as shown in Fig. S2a. The few-layer flake has a quasi-triangular shape, and its height of about 4.15 nm corresponds to the five layers (for monolayer thickness of ~0.8 nm)[11,12]. The transmission electron microscope (TEM) were used to characterize the microstructure and chemical composition of the GaSe layer. Figure S2b displays a typical TEM image of the few-layer GaSe nanosheets, and the corresponding element mapping images shown in Fig. S2c and Fig. S2d indicate uniformly distributed Ga and Se elements, confirming uniform chemical composition along the nanosheet with a Ga/Se atomic ratio of 1:1. Moreover, from Fig. S2e, the electron diffraction image of the GaSe nanosheet also shows uniform layer and perfect hexagon-like crystalline lattice structure in plane.

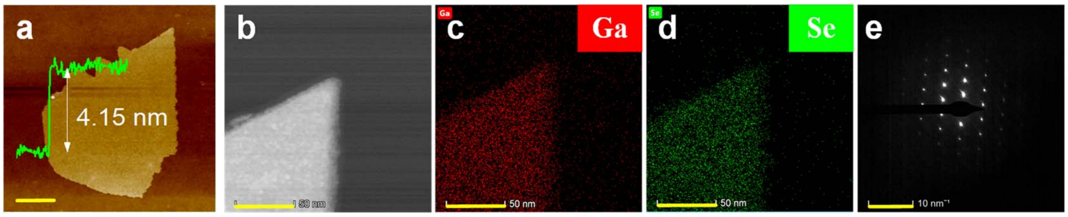

Figure S2 (**a**) Atomic force microscope (AFM) image of the GaSe layer employed to integrate with microfibre, revealing about 4.15 nm thickness. (**b**) Typical transmission electron microscope (TEM) image of triangular edge of the GaSe layer. (**c, d**) Ga and Se element maps, (**e**) electron diffraction image of the corresponding GaSe layer in (**b**), showing uniform layer and perfect hexagon-like crystalline lattice structure in plane. Scale bars: a: 2 μm; b-d: 50 nm; e: 10 nm^{-1}.

Measurement setup of SHG and SFG signals from the GaSe-integrated microfibre

Figure S3 schematically depicts the experimental arrangement for measuring SHG and SFG of the GaSe-integrated microfibre. Different light sources including a picosecond pulsed laser, two CW lasers (a narrowband tunable laser around 1550 nm and a distributed feedback laser at 1310 nm) are selectively coupled into the GaSe-integrated microfibre to excite the SHG or SFG. After the output port of the device, the frequency up-converted signals together with the propagating pump light are separated by a free-space filtering system, which are then examined by a spectrometer mounted with a cooled silicon CCD. In the implementation of the SFG process, the two CW lasers at the wavelengths around 1550 nm and 1310 nm are

combined via a 1310/1550 WDM to input into the GaSe-integrated microfibre.

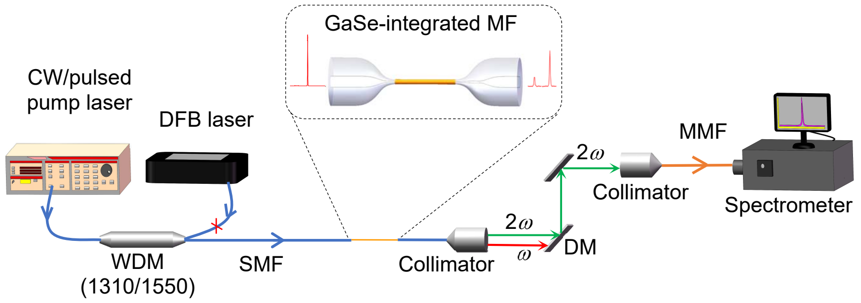

Figure S3 Schematic diagram of measurement arrangement for measuring the SHG and SFG signals in the GaSe-integrated microfibre. SMF: single-mode fibre; MMF: multi-mode fibre; MF; microfibre; DFB laser: distributed feedback laser; WDM: wavelength division multiplexer; DM: dichroic mirror.

SHG signals from microfibres with different diameters

Since the diameter and shape of the microfibre have a great effect on the SHG signals, we first used a pulsed laser with a high peak-power to tentatively examine the SHG signal from the GaSe-integrated microfibre and choose the optimal diameter. When the larger diameter (~4 μm) microfibre is used, the SHG signal is very weak and difficult to be detect stably. When the smaller diameter (~2.5 μm) microfibre is used, the SHG signal is much weaker (~38 times) than that from the ~3.1 μm microfibre, as shown in Fig. S4. Therefore, the power- and wavelength-dependence of the CW-pumped SHG and SFG are investigated under the optimal diameter.

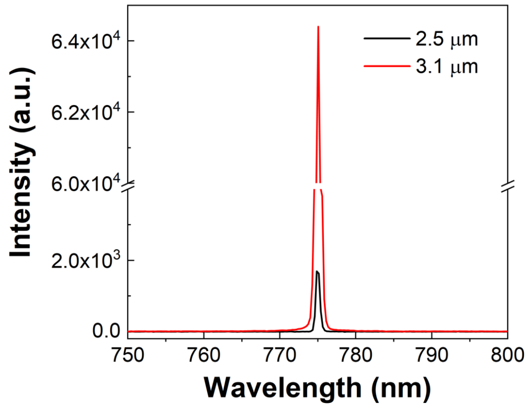

Figure S4 Spectra of SHG signal from GaSe-integrated microfibres with different diameters.

Measurement setup of side-scattered SHG signal

The SHG signals side-scattered from the GaSe-integrated microfibre surface are scanned and collected using a vertically coupled microscope, as schematically illustrated in Fig. S5. In the measurement, the GaSe-integrated microfibre was mounted on a 3D micro-translation platform, the SHG was pumped by the evanescent field of the microfibre, and the pumped light source is a picosecond laser (PriTel FFL-20MHz). The objective lens of the microscope system is a near-infrared anti-reflective one with a 50× magnification and a numerical aperture of 0.42. A visible white light source and a silicon-based charge coupled device (CCD) are used to track the position of microfibre before the SHG signal collection. The shutter is closed during the SHG signal collection for avoiding the disturbance of background white light. The side-

scattered SHG signals and pump light signal from the GaSe-integrated microfibre are vertically collected by the objective lens and spectrometer via a short-pass dichroic mirror (DM) with cut-off wavelength of 1000 nm. By precisely adjusting the position of GaSe-integrated microfibre (the inset of Fig. S5) along the fibre axis with the translation stage, the SHG signal from different locations of the device can be obtained. The measured results are shown in Fig. 3(b) of the maintext.

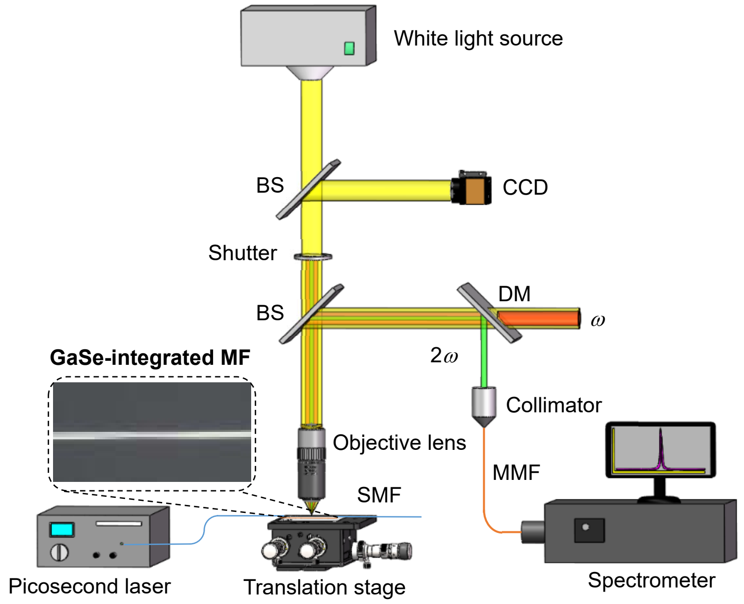

Fig. S5 Measurement setup of the SHG signals sidewise-scattered from the GaSe-integrated microfibre. The inset shows the optical microscope image of GaSe-integrated microfibre. BS: beam splitter; DM: dichroic mirror; MF: microfibre; SMF: single-mode fibre; MMF: multi-mode fibre; CCD: charge coupled device.

Pump-power dependence of the SHG at 1310 nm

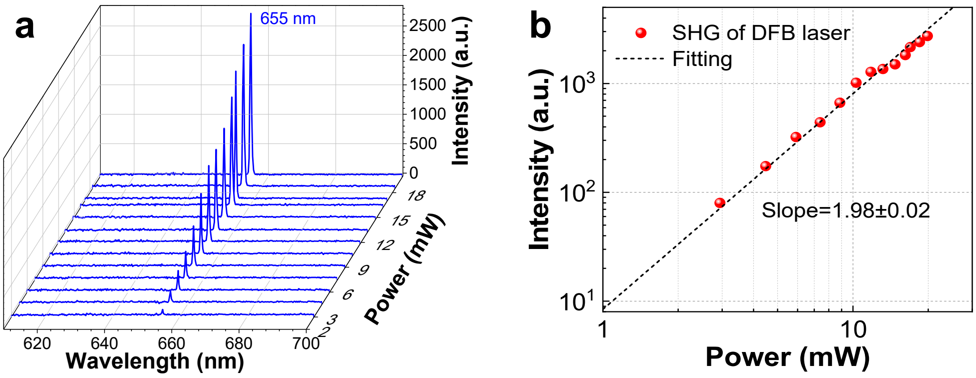

Fig. S6 (**a**) Measured spectral evolution of SHG pumped by a DFB laser of 1310 nm and varied the incident power from 0 to 20 mW with the help of a tunable attenuator, and (**d**) corresponding log-log plot with a fitting slope of 1.98.

By using the experimental system shown in Fig. S3, at another telecom wavelength 1310 nm, the incident power of the DFB laser was varied by adjusting a tunable attenuator, and then we obtained a similar power dependence with that pumped at the 1550 nm. The detailed results are shown in Figs. S6a and S6b, and the fitting slope of log-log plot is 1.98±0.02, also following the predicted quadratic dependence. Nevertheless, the output SHG intensity is twice

stronger than that pumped at 1550 nm under the same pump power.

Estimation of SHG conversion efficiency

In the measurement system shown in Fig. S3, when the coupling efficiency of 80% via the WDM into the microfibre device, and the reflectance (~90%) of the dichroic mirror and coupling efficiency (~60%) via collimator into the multi-mode fibre and spectrometer/ photomultiplier tube are taken in account, the actual pump and SHG powers are around 8 mW and 185 pW, respectively. Then, the SHG conversion efficiency is estimated as 185 pW/(8 mW)^2=2.9×10^{-4} %/W. This efficiency is more than four orders of magnitude higher than those reported in previous works. For instance, in a similar microfibre with a length of 10 mm and a diameter around 0.7 μm [13], the SHG conversion efficiency is 4.2×10^{-8} with a pump of 90 W, 5 ns, 1.55 μm pulses, and the normalized efficiency is calculated as 4.7×10^{-8} %/W. In Ref. 14, for a 2.5 cm long chalcogenide–tellurite hybrid optical fibre, pumped by a ~200 fs pulsed laser, the SHG conversion efficiency is in the order of 10^{-8} at the peak power of 6.48 kW, and the corresponding normalized efficiency is 1.5×10^{-10} %/W. In Ref. 15, for a 15 cm long silica photonic crystal fibre, pumped by a 120 fs pulsed laser, the maximum conversion efficiency is 1.6×10^{-6} at the peak power of 60 kW, and the corresponding normalized efficiency 4.1×10^{-9} %/W when taking the coupling efficiency of 65% in account. Therefore, integrating GaSe nanoflakes onto microfibres could be considered as an effective way to realize high-efficiency SHG in optical fibres leveraging GaSe's strong second-order nonlinearity.

Determination of stability and thresholds of the frequency conversion

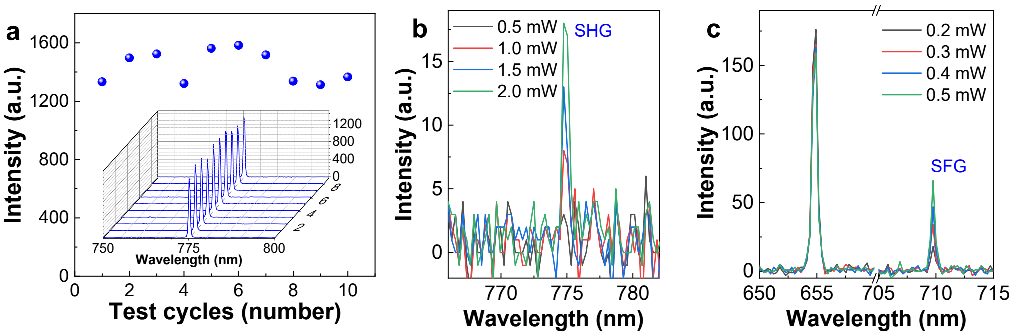

Figure S7 Determinations of the repeatability and the limit of pump power for SHG and SFG processes. (**a**) Different SHG signals measured over 2 hours when pumped by the Pump-2 at 1550 nm. (**b**) Spectra of SHG signals only pumped by Pump-2 with different microwatt-level powers. (**c**) Spectra of SFG signals pumped by a stable Pump-1 and power-varied Pump-2 with different sub-microwatt-level powers.

To examine the stability and repeatability of the frequency conversion, we detected the output SHG signal of GaSe-integrated microfibre at different times, and the corresponding spectra are shown in the inset of Fig. S7a. Multiple measurement results show that the SHG intensity plateaued at around 1400 (a.u.) over 2 hours. In the detection process, since the device is exposed to air, the fluctuation of the signal within ~9% in Fig. S7a could be caused by the disturbance of air flow, dust capture and the instability of measurement configuration. The stability could be further improved by effective coating package for engineering application. In addition, we determined the thresholds of pump power for stable and detectable SHG and SFG signals by reducing the pump power. For the independently pumped

SHG, the pump power was tuned by steps of 0.5 mW between 0.5 and 2 mW, the results are shown in Fig. S7b. We observed the SHG signal when the pump power could be reduced down to less than 1 mW relying on the strong optical nonlinearities of GaSe layer. For the simultaneously pumped SFG, by fixing one lasers at 17 mW and reducing another from 0.5 mW to 0.2 mW, we obtained a decreasing SFG signal as well as a stable SHG, as depicted in Fig. S7c. The SFG signal could be observed at sub-milliwatt-level pump power all the time. From the results, we can confirm that the limits of the pump power for SHG and SFG are less than 1 mW and ~0.2 mW, respectively. Moreover, the microfibre, space-to-fibre coupling and other insertion losses are taken in account, totally tested by ~4 dB (see Fig. S1), much lower power even tens of microwatts can generate above detectable frequency conversion signals.

References

1 Gan, X. T. *et al.* Graphene-assisted all-fibre phase shifter and switching. *Optica* **2**, 468-471, doi:10.1364/OPTICA.2.000468 (2015).

2 Guo, J. *et al.* Doped GaSe crystals for laser frequency conversion. *Light: Science & Applications* **4**, e362, doi:10.1038/lsa.2015.135 (2015).

3 Zhou, X. *et al.* Strong second-harmonic generation in atomic layered GaSe. *Journal of the American Chemical Society* **137**, 7994-7997, doi:10.1021/jacs.5b04305 (2015).

4 Kumar, N. *et al.* Second harmonic microscopy of monolayer MoS_2. *Physical Review B* **87**, 161403, doi:10.1103/PhysRevB.87.161403 (2013).

5 Gan, X. T. *et al.* Microwatts continuous-wave pumped second harmonic generation in few- and mono-layer GaSe. *Light: Science & Applications* **7**, 17126, doi:10.1038/lsa.2017.126 (2018).

6 Mao, D. *et al.* Erbium-doped fibre laser passively mode locked with few-layer WSe_2/$MoSe_2$ nanosheets. *Scientific Reports* **6**, 23583, doi:10.1038/srep23583 (2016).

7 Jie, W. *et al.* Layer-dependent nonlinear optical properties and stability of non-centrosymmetric modification in few-layer GaSe sheets. *Angewandte Chemie International Edition* **54**, 1185-1189, doi:10.1002/anie.201409837 (2015).

8 Lacroix, S., *et al*. Tapered monomode optical fibres: understanding large power transfer. *Applied Optics* **25**, 4421-4425, doi:10.1364/AO.25.004421 (1986).

9 Li, W. *et al.* Ultrafast all-optical graphene modulator. *Nano Letters* **14**, 955-959, doi:10.1021/nl404356t (2014).

10 Cassidy, D. T., Johnson, D. C. & Hill, K. O. Wavelength-dependent transmission of monomode optical fibretapers. *Applied Optics* **24**, 945-950, doi:10.1364/AO.24.000945 (1985).

11 Tang, Y., Mandal, K. C., McGuire, J. A. & Lai, C. W. Layer- and frequency-dependent second harmonic generation in reflection from GaSe atomic crystals. *Physical Review B* **94**, 125302, doi:10.1103/PhysRevB.94.125302 (2016).

12 Zhou, Y. *et al.* Epitaxy and photoresponse of two-dimensional GaSe crystals on flexible transparent mica sheets. *ACS Nano* **8**, 1485-1490, doi:10.1021/nn405529r (2014).

13 Gouveia, M. A. *et al.* Second harmonic generation and enhancement in microfibres and loop resonators. *Applied Physics Letters* **102**, 201120, doi:10.1063/1.4807767 (2013).

14 Cheng, T. *et al.* Widely tunable second-harmonic generation in a chalcogenide-tellurite hybrid optical fibre. *Optics Letters* **39**, 2145-2147, doi:10.1364/OL.39.002145 (2014).

15 Yuan, J. *et al.* Generation of second-harmonics near ultraviolet wavelengths from femtosecond pump pulses. *IEEE Photonics Technology Letters* **28**, 1719-1722, doi:10.1109/LPT.2016.2530744 (2016).
